# Establishing standards for Yonsei point in a White South African population for the treatment of gummy smile

**DOI:** 10.1002/cre2.735

**Published:** 2023-04-18

**Authors:** Brandon Booysen, René H. Baron, André Uys

**Affiliations:** ^1^ Department of Anatomy, Faculty of Health Sciences University of Pretoria Arcadia South Africa

**Keywords:** botox injections, hyperactive lip elevator muscles, injection landmark radii, Yonsei point

## Abstract

**Introduction:**

The purpose of this study is to establish the efficacy of Yonsei point in the treatment of a gummy smile in a White South African population. The accurate surface anatomy criteria in relation to the underlying musculature for the administration of Botulinum toxin injections in the treatment of gummy smile was determined.

**Materials and Methods:**

Nineteen (10 males and 9 females) cadavers were selected for facial dissection. Facial profile photographs were taken before and after dissection. The before and after photographs were overlayed to determine where the pin positions should be on the dissected cadaver to determine the Yonsei point. The levator labii superioris (LLS), LLS alaeque nasi (LLSAN), zygomaticus minor, and zygomaticus major muscles were measured using a protractor and ruler, which accounted for the manual measurements. Digital measurements were measured by importing dissected images into ImageJ. Circles with a 2 cm diameter (1 cm radius) were constructed to determine whether the Yonsei point could successfully influence muscles fibers.

**Results:**

Digital and manual measurements show comparable results with high correlation and reliability. Results showed that the White South African population had narrower facial musculature angles as compared with the Korean population.

**Conclusion:**

Based on the selected sample, the Yonsei point is an ineffective injection site for the successful treatment of gummy smile in a White South African population.

## 1 INTRODUCTION

Excessive gingival tissue presentation during smiling, colloquially known as gummy smile, is a feature of the smile in which more than 4 mm of gingival tissue is on display (Hwang et al., 2009). Gummy smile is relatively common, affecting up to 29% of the population with females being more prominently affected (Dym & Pierre, 2020; Tjan et al., 1984). Often this feature is considered unesthetic and detracts from the smile (Dong et al., 1999). Factors that may contribute to the appearance of the gummy smile are thought to be as a result of skeletal, gingival, and muscular anomalies (Moura et al., 2017). These anomalies are related to the abnormal gingival movement away from the crown of the teeth (altered passive eruption), overeruption of teeth (dentoalveolar extrusion), excessive maxillary growth in the vertical plane (vertical maxillary excess), and short or hyperactive lip elevator muscles (Mostafa, 2018). Orthodontists have previously tailored their treatment to target hard tissue structures such as the maxilla. As a result, very minimal treatment and attention is given to hyperactive lip elevator muscles (Moura et al., 2017). Although orthognathic surgery is invasive, this treatment option is used for patients with gummy smiles that are not mainly caused by hyperactive lip elevator muscles. The treatment for hyperactive lip elevator muscles is minimally invasive and can be performed with administration of Botulinum toxin (Botox) injections into the muscles (Mostafa, 2018).

The muscles that are responsible for the elevation of the lip are the levator labii superioris (LLS), LLS alaeque nasi (LLSAN), zygomaticus minor (ZMi), and zygomaticus major (ZMj) (Agur et al., 2019). Injection of Botox intramuscularly causes the cleavage of synaptosome‐associated protein (SNAP‐25). As a result, acetylcholine is blocked from exocytosis and causes partial denervation of the muscles (Mostafa, 2018). This effectively causes the lip elevator muscles to depress during smiling and helps to conceal the gum.

Several studies have been conducted to determine the efficacy and effectiveness of Botox injections at certain points in the face to treat a gummy smile (Mazzuco & Hexsel, 2010; Polo, 2008; Sucupira & Abramovitz, 2012). The safest and most effective site for Botox treatment as proposed by Hwang et al. (2009) is located in the face at a place known as “Yonsei point.” The administered dose of the Botox injection should be based on the severity of the gummy smile; however, a maximum of 5 IU with initial injections is a safe approach to treatment (Duruel et al., 2019). Yonsei point is an area lateral to the nose in which the LLS, LLSAN, and ZMi all pass through a triangular space created by three points. The three points are the lateral point of the ala of the nose, midpoints of nasolabial fold in between the ala and commissure, and the maxillary point about one‐quarter between the ala and tragus of the ear (Moura et al., 2017). With one Botox injection at this point, the LLS, LLSAN, and ZMi are successfully targeted and opposes the need to administer one injection into all three muscles.

As established above, Yonsei point is a generalized point used by clinicians to determine the site of Botox injections to effectively treat gummy smile. However, due to the vast number of variations between populations and ancestry groups around the world, the generalized Yonsei point might not be accurate for every population group. This is due to slight facial morphological differences between population groups. Results show that the average Korean male and female have a broader and more prominent malar and zygomatic region compared with Houstonian White faces (Kim et al., 2016). Other differences found were more protrusion in the glabella, nasion, and pogonion regions. This suggests that different population groups might have slightly different Yonsei points, although reference data for other populations is limited. Therefore, it is important to establish population standards for the Yonsei point to ensure that the treatment thereof is safe and effective.

In South Africa, there is limited research and treatment standards regarding a gummy smile. Establishing South African standards for the location of the Yonsei point is vital in ensuring safe and effective treatment for a gummy smile using Botox injections.

Thus, the aim of the study is to determine the accurate surface anatomy criteria for the administration of Botox injections to treat a gummy smile in the South African population. The objectives of this study are to establish accurate surface landmarks pertaining to the lip elevator muscles based on muscle morphology, establish a point (Yonsei point) in which the administered Botox has the greatest effect on the lip elevator muscles to treat a gummy smile, and, lastly, to determine the variability between lip elevator muscles in the South African population so that Botox injections can be more safely and accurately administered.

## 2 MATERIALS AND METHODS

### 2.1 Materials

Thirty‐eight hemifaces from 19 adult, White South African embalmed cadavers (10 males and 9 females) from the Department of Anatomy, University of Pretoria, were included. All cadavers were embalmed with a 4% formaldehyde solution. The White South African population are historically descendants from German, Dutch, French, and British populations (Davenport & Saunders, 2000). A convenience sampling method was applied to include a representative sample of male and female cadavers. Cadavers with intact skin overlying the facial muscles, without a history of pathology and facial surgery, were included. This study falls under the auspices of the National Health Act 61 of 2003. Ethical clearance for the use of cadavers was obtained from the Research Ethics Committee of the Faculty of Health Sciences, University of Pretoria (173/2022).

### 2.2 Methods

**Figure 1:**
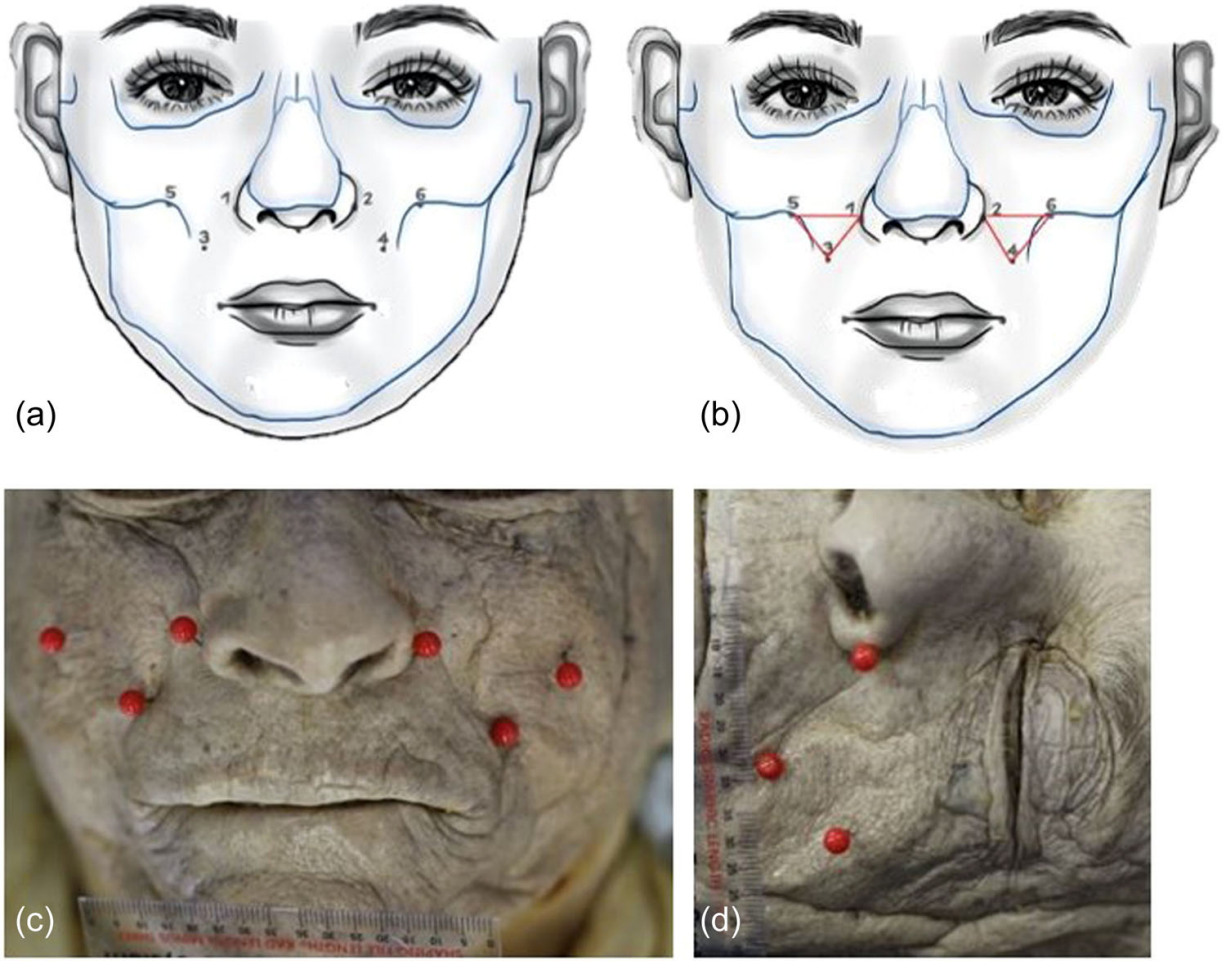
(a) Pin positions 1– 6, (b) constructed triangle, (c) frontal photograph of cadaver with pin positions, (d) perpendicular photograph (to the surface of the pins) of the cadaver showing pin positions.

Before dissection commenced, pins were placed at various surface landmarks of the face. These identification points represent surface landmarks for individual muscle Botox injection sites (Figure 1 and Table 1).

**Table 1:** Definition of the surface landmarks for Botox injection sites.

Landmark	Definition
Pins 1 and 2	Lateral point of ala of the nose
Pins 3 and 4	Midpoints of nasolabial fold between the ala and commissure
Pins 5 and 6	Maxillary point about one‐quarter between the ala and tragus of the external ear
Pin 7	Soft tissue subnasale
Pins 8 and 9	Commissure
Pin 10	Soft tissue pogonion
Pin 11 and 12	Lateral chin point located 2 cm on either side of the pogonion

From these pin positions, a triangular area was constructed using pin positions 1 and 2, 3 and 4, and 5 and 6 on both sides of the face (Figure 1b). As previously stated, these pin positions also represent injection landmarks/sites for the regular treatment of gummy smile using Botox injections. Frontal as well as perpendicular photographs of the cadavers on both sides of the face were taken before dissection showing the constructed triangle (Figure 1c,d). An indicating ruler was placed at skin level for standard reference scale purposes.

**Figure 2:**
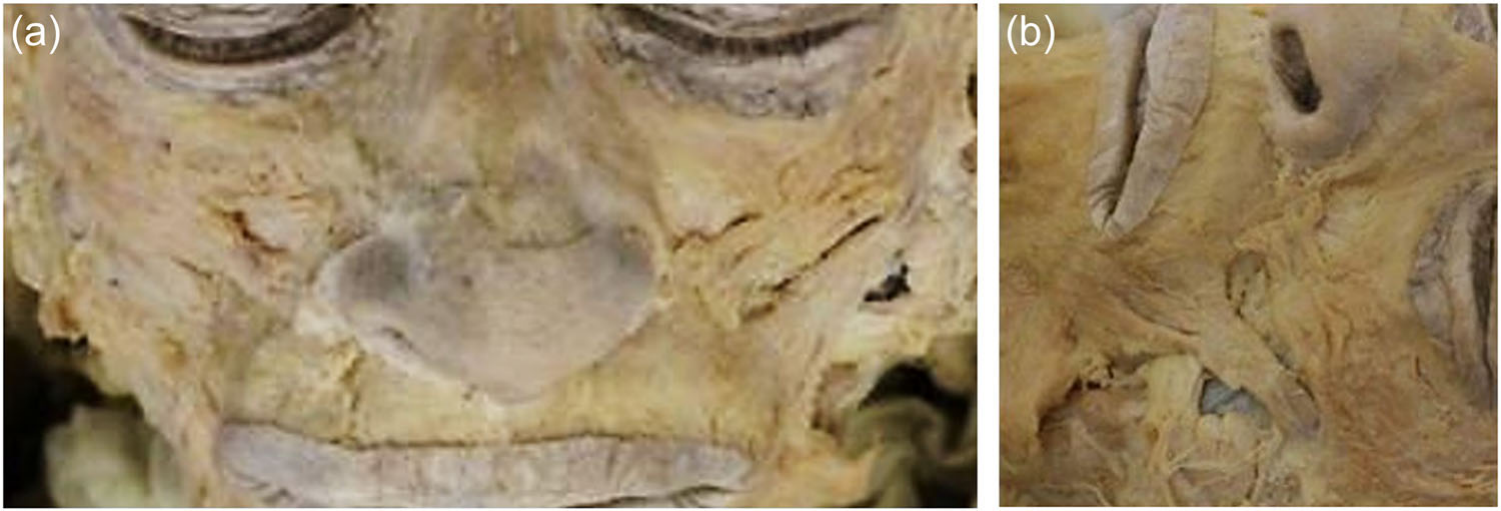
(a) Frontal and (b) perpendicular photograph of a dissected cadaver.

A midline incision was made between the soft tissue points of the glabella, subnasale, and pogonion. After the incision along the midline, the LLS, LLSAN, ZMj, and ZMi were dissected carefully, exposing the muscle fiber orientation. Photographs were taken after dissection from the frontal and perpendicular angle to show the muscle fiber orientation and direction (Figure 2a,b).

**Figure 3:**
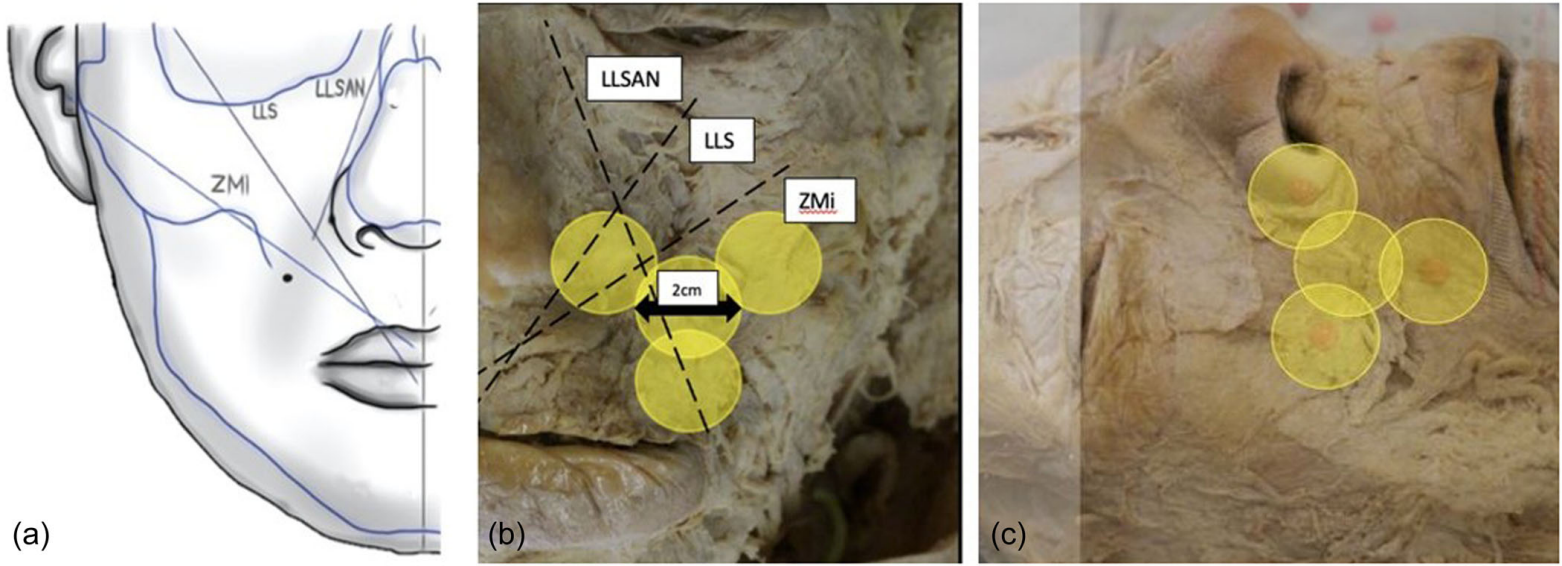
(a) Muscle vector directions in relation to the midline of the face, (b) muscle vector directions and Botox spread (2 cm) on dissected cadaver, (c) overlaid photograph showing Botox spread at Yonsei point and the individual injection landmarks.

Using a protractor and ruler, manual measurements were taken on all 38 hemifaces. This was done to compare whether there were any significant differences between the manual and digital measured angles. Dissected pictures were imported to the computer image programming system ImageJ (version 1.53t manufactured by NIH image). Using the angle tool on ImageJ, a vector was constructed on the photographs from the origin and insertion of the lip elevator muscles. The vector represents the center of each muscle fiber bundle. The midline was used as a point of reference to measure the angle created between each vector of the lip elevator muscles. Depending on muscle fiber direction in relation to the midline, angle values were measured being either positive or negative. In Figure 3a, Measurement 1 represents the angle between the ZMi and facial midline, Measurement 2 represents the angle between the LLS and the facial midline and Measurement 3 represents the angle between the LLSAN and the facial midline. The LLSAN measurements will be negative due its vector direction in relation to the facial midline.

Once the angle measurements were complete, undissected and dissected cadaver images were imported to Microsoft Office Word version 2021. Using the overlay function, corresponding undissected and dissected images were overlayed showing the constructed triangle with the landmark injection points and the direction of muscle fibers. Circles were constructed on each injection landmark and at the Yonsei point in the center of the triangle. These circles are known as the Botox effective range or the radius of effectiveness and illustrates how far away the Botox can spread to effect muscles from its initial injection point (Figure 3b,c). The Botox effective range was adjusted to 2 cm; therefore, circles with a radius of 1 cm were drawn as stated above. All measurements were calibrated according to the indicating ruler.

**Figure 4:**
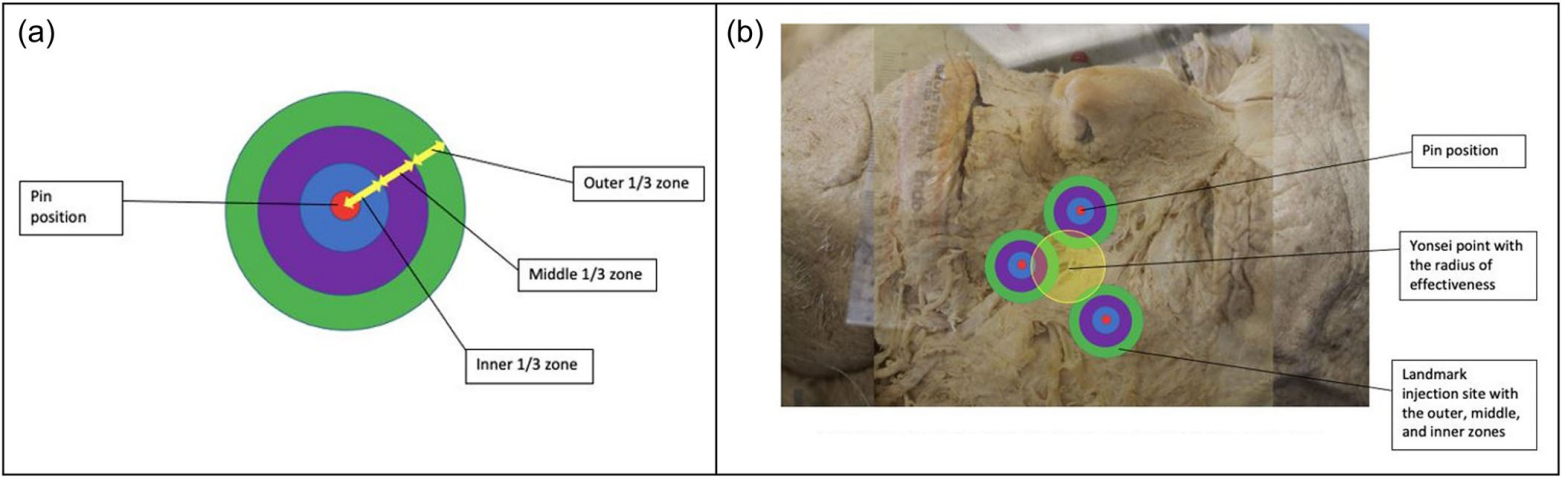
(a) Injection landmark zones and (b) overlap of Yonsei point radius with the injection landmark radii.

For Botox injections to be effective in causing muscle paralysis when injected at the Yonsei point, the radius of effectiveness would need to cross as close to the center of the landmarks (injection points) as possible. The center of the landmarks (denoted by the red pins in Figure 4a) is thought to be the place where most muscle fibers in the area would be successfully affected by a Botox injection. Each landmark would have its own radius of effectiveness if an injection was administered. Therefore, it was important to establish how far the Yonsei point injection radius would spread and whether it would be enough to successfully effect the muscles in the area. The radius of Botox spread of each injection landmark was divided into three areas/zones surrounding the pin. The zones were divided into thirds: the inner one‐third, middle one‐third, and outer one‐third (Figure 4a). For the Yonsei point to be a viable injection site, its radius of effectiveness would need to pass through the inner one‐third of the landmark injection radii. The prevalence of the overlaps of circular areas of each landmark was determined and recorded (Figure 4b).

### 2.3 Statistics

Statistical analyses were conducted in SPSS Statistics 28.0.1.0(142) (IBM) to express the distribution of each muscle according to the vector directions in relation to the midline of the face. Basic descriptive statistics was used to determine parameters such as minimum, maximum, mean, median, and quartiles. Independent *t* tests were used to assess the differences between the male and female specimens, as well as between the left and right measurements. The significance level was adjusted to *p* ≤ .05. The correlation between digital and manual measurements were evaluated by determining the inter‐class correlation (ICC) values for all measurements. Interobserver repeatability was assessed by re‐evaluating 10 of the cadaver measurements via ICC.

## 3 RESULTS

Table 2 shows the angle measurements taken digitally and manually for the LLS, LLSAN, ZMi, and ZMj muscles for the right and left side for each male and female cadaver.

**Table 2:** Digital and manual angle in degrees (°) measurements for the lip elevator muscles in males and females.

Muscle	LLS Left				LLS Right			
Sex	Males		Females		Males		Females	
Measurement	Digital	Manual	Digital	Manual	Digital	Manual	Digital	Manual
Mean	30.4	30.7	30.1	28.2	30.4	30.7	30.0	29.2
SD	3.1	3.6	3.7	3.6	3.1	3.6	3.8	3.3
Minimum	25.1	26.0	27.0	22.0	25.1	26.0	25.2	25.5
Maximum	36.7	35.0	36.7	32.5	36.7	35.0	37.0	35.0

Muscle	LLSAN left				LLSAN right			
Sex	Males		Females		Males		Females	
Measurement	Digital	Manual	Digital	Manual	Digital	Manual	Digital	Manual
Mean	−14.4	−16.3	−13.6	−15.0	−13.0	−14.7	−11.6	−13.1
SD	2.0	3.4	3.0	2.7	1.8	2.0	3.3	2.8
Minimum	−16.7	−24.0	−17.2	−19.5	−15.2	−18.0	−15.3	−15.5
Maximum	−10.6	−11.5	−8.1	−11.5	−9.4	−10.5	−5.6	−9.5

Muscle	ZMi Left				ZMi Right			
Sex	Males		Females		Males		Females	
Measurement	Digital	Manual	Digital	Manual	Digital	Manual	Digital	Manual
Mean	46.0	52.1	45.5	54.6	45.2	55.2	43.9	57.7
SD	3.5	5.6	5.9	5.6	6.7	9.1	6.5	6.8
Minimum	40.1	41.5	34.3	44.5	37.5	43.0	35.3	48.0
Maximum	52.1	60.5	55.3	64.0	58.7	62.0	57.5	71.5

Muscle	ZMj Left				ZMj Right			
Sex	Males		Females		Males		Females	
Measurement	Digital	Manual	Digital	Manual	Digital	Manual	Digital	Manual
Mean	51.5	54.5	47.6	54.6	50.3	58.2	49.5	58.8
SD	5.2	5.6	4.6	5.9	3.4	5.2	8.4	4.7
Minimum	43.3	48.0	40.8	45.0	45.3	44.5	38.1	50.0
Maximum	59.0	66.5	53.9	60.5	55.4	63.5	63.8	65.5

Abbreviations: LLS, levator labii superioris; LLSAN, levator labii superioris alaeque nasi; ZMi, zygomaticus minor; ZMj, zygomaticus major.

Tables 3 and 4 depict the overlapping of the Yonsei point with the injection landmark radii.

**Table 3:** Overlap of Yonsei point radii with the injection landmark radii for males.

	Lateral point of ala nose landmark			1/4 between tragus and ala landmark			Nasolabial fold landmark		
Male cadavers	Outer 1/3	Middle 1/3	Inner 1/3	Outer 1/3	Middle 1/3	Inner 1/3	Outer 1/3	Middle 1/3	Inner 1/3
101‐ Left		x		x					x
101‐ Right			x			x			x
103‐ Left		x			x		x		
103‐ Right		x			x				x
105‐ Left	x			–	–	–	x		
105‐ Right	–	–	–	x					x
109‐ Left		x		x				x	
109‐ Right		x			x				x
111‐ Left			x		x				x
111‐ Right		x			x				x
113‐ Left		x			x			x	
113‐ Right		x			x				x
117‐ Left		x			x			x	
117‐ Right		x			x				x
119‐ Left		x			x			x	
119‐ Right	x				x				x
201‐ Left		x			x			x	
201‐ Right		x		x					x
221‐ Left		x		x					x
221‐ Right	x			x					x
Total	3	14	2	6	12	1	2	5	13

*Note*: X represents where Yonsei point overlapped with the injection landmark radii on each cadaver. A dash represents where no overlapping of Yonsei point with the injection landmark radii at all. The colours in table represent the overlapping injection sites in Figure 6.

**Table 4:** Overlap of Yonsei point radii with the injection landmark radii for females.

	Lateral point of ala nose landmark			1/4 between tragus and ala landmark			Nasolabial fold landmark		
Female cadavers	Outer 1/3	Middle 1/3	Inner 1/3	Outer 1/3	Middle 1/3	Inner 1/3	Outer 1/3	Middle 1/3	Inner 1/3
102‐ Left			x			x			x
102‐ Right			x			x			x
104‐ Left			x		x				x
104‐ Right		x			x				x
110‐ Left		x			x			x	
110‐ Right		x			x			x	
112‐ Left			x			x			x
112‐ Right			x			x			x
118‐ Left	x			x			x		
118‐ Right		x			x				x
120‐ Left		x			x				x
120‐ Right		x				x			x
202‐ Left		x			x				x
202‐ Right	x				x				x
210‐ Left		x			x			x	
210‐ Right		x			x				x
218‐ Left		x		x				x	
218‐ Right	x			x					x
Total	3	10	5	3	10	5	1	4	13

*Note*: X represents where Yonsei point overlapped with the injection landmark radii on each cadaver. A dash represents no overlapping of Yonsei point with the injection landmark radii at all. The colours in table represent the overlapping injection sites in Figure 6.

Intra‐class coefficient values (ICC) for digital versus manual were 0.963 for all measurements combined and the inter‐rater reliability had a coefficient of 0.957.

## 4 DISCUSSION

The digital and manual measurements showed a high degree of agreement (Bobak et al., 2018). Manual or digital measurements can be successfully used to determine the angle measurements for facial muscles.

In this study, it was found that there was a lack of efficacy of the established Yonsei point in the white South African population when comparing the results with the literature.

Differences in the angle measurements between the Korean and the White South African population were noted with the Korean population demonstrating larger muscle angles (Moura et al., 2017). A difference of around 10° between the Korean and South African population was found. This data shows that the average Korean male and female have a broader and more prominent malar and zygomatic region compared to the study population.

**Figure 5:**
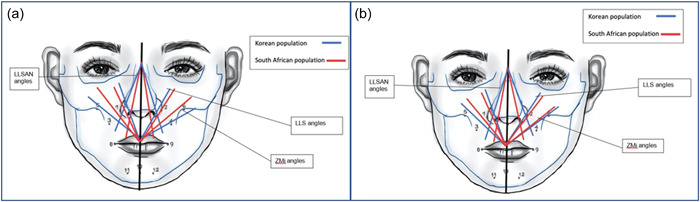
(a) The differences between the muscle angles in the male Korean and male South African population. (b) The differences between the muscle angles in the female Korean and female South African population.

The Korean population had a larger LLSAN angle, whereas the White South African population showed a larger LLS angle. The difference in angle measurements may explain why the Yonsei point is not a viable single Botox injection site (Figure 5). For Botox to be effective, a spread of about 1–2 cm around the injection site is necessary. Injections at the wrong site can lead to partial relaxation of other facial muscles that do not require treatment (Al Wayli, 2019). Only five hemifaces demonstrated that the Yonsei point is a viable injection site for the White South African population.

The lateral ala landmark showed a high degree of overlap in the middle one‐third zone. This was more prevalent in females and can be explained by the smaller facial dimensions.

Lakhiani and Somenek (2019) explored facial morphological differences based on sex and affirmed that males have a generally larger interzygomatic distance.

In males specifically, the tragus injection landmark showed the least affinity for the efficacy of the Yonsei point for the inner third radii. In females, both the lateral point of the ala of the nose and the tragus landmark showed poor inner third overlap. However, t it can be concluded that both landmarks are equally uneffaced by Yonsei point.

The results of this study showed that Yonsei point, as defined in literature, is not a viable site for Botox injection in the White South African population. It is proposed that in the White South African population, two separate Botox injections might be more effective in treating gummy smile. The nasolabial fold had the most affinity for Yonsei point in terms of overlap in the inner one‐third zone of the injection landmark. The differences seen in the efficacy at the lateral point of the ala of the nose landmark between the Korean and White South African population may be due to differences in ala size. Zaidi et al. (2017) found that the distance between the ala of the nose is greater in individuals of West African, South Asian, and East Asian ancestries as compared with individuals of European ancestry. This could suggest that the injection landmarks are closer together in the Korean population as compared with the White South African population.

**Figure 6:**
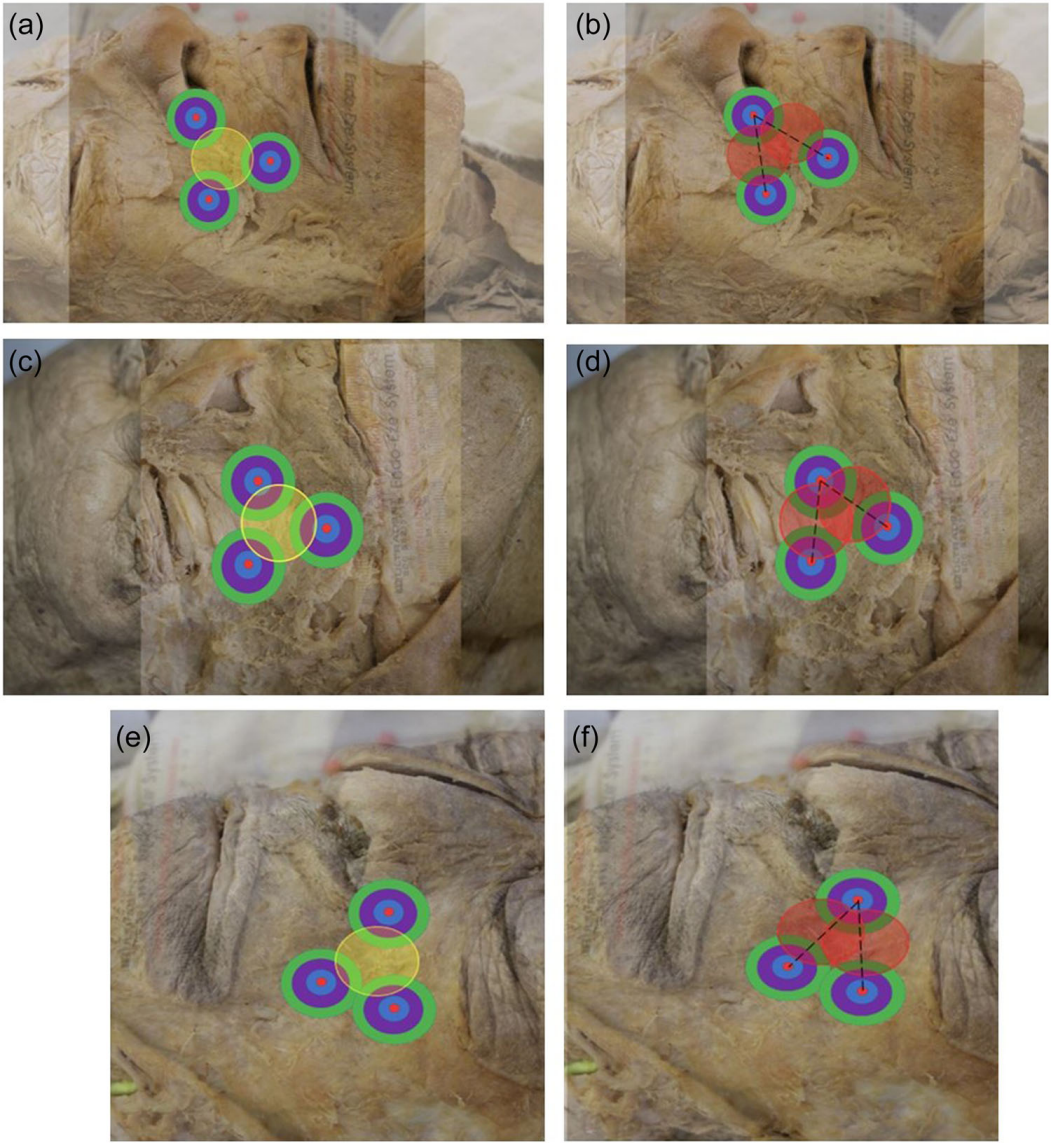
(a, c, e) Yonsei point (yellow) with overlap of individual injection sites and (b, d, f) corresponding cadaver with new proposed injection landmarks (red) located at the center of the planes running between the specific injection landmarks.

To improve and maximize the treatment of gummy smile using Botox injection in the White South African population, it is proposed that two injection landmarks might be more effective as demonstrated in study examples (Figure 6).

Limitations of this study are that all cadavers used were White South African individuals of European descent and the sample size was small. In future research, it would be beneficial to increase the sample size and compare findings with other population groups. This study should be repeated using fresh cadaver specimens, as the effect of embalming on facial muscle dimensions are unknown. Due to the embalming process, cadavers may have distorted facial muscles, which could affect results. The study population consisted of older individuals and age‐related changes might also influence the results of the study. All the muscles investigated are muscles of facial expression and the dentition status should have a minimal effect on the muscle tone and angulation. Future studies should compare the effect of different dentate statuses. Other limitations include the inability to assess muscle activity and the smile configurations in the embalmed cadavers (Moura et al., 2017). Lastly the inability to investigate the effectiveness of Botox spread on the cadavers is a limitation.

Based on the sample, the Yonsei point is an unlikely injection site for a viable and successful treatment of gummy smile in the white South African population. This is due to the musculature angle differences between the Korean population and the White South African population. This means that the Yonsei point in its proposed position as determined by Hwang et al. (2009) does not effectively target the muscles in all the regions of the injection landmarks when applied to a White South African population. As stated in the discussion, the only injection landmark that is adequately targeted by the Yonsei point is the Nasolabial fold injection point. The other injection landmarks do not have sufficient overlaps in the inner one‐third radius; therefore, the Yonsei point as defined in the literature, would suffice as a sufficient area to target all three injection landmarks. It is therefore proposed that two injection points should be used to successfully target all the injection landmarks in a White South African population. The two proposed injections points are at the midpoint on the plane between the lateral point of the ala of the nose and the nasolabial fold, as well as at the midpoint on the plane between the lateral point of the ala of the nose and the tragus landmark. Although these are proposed landmarks, further research would need to be conducted to evaluate its viability, repeatability and validity.

## AUTHOR CONTRIBUTIONS

Brandon Booysen and André Uys made substantial contributions to the conception and design of the study. Brandon Booysen was responsible for the acquisition of data. Brandon Booysen and André Uys analyzed and interpreted the data. Brandon Booysen, André Uys, and René H. Baron were involved in the drafting of the manuscript, revision of the manuscript, and final approval of the version to be published. All the authors are accountable for all aspects of the work and ensured the accuracy and integrity of the work.

## CONFLICT OF INTEREST STATEMENT

The authors declare no conflict of interest.

## ETHICS STATEMENT

The Department of Anatomy, University of Pretoria, is the custodian for the human embalmed cadavers used. This study falls under the auspices of the National Health Act 61 of 2003. Ethical clearance was granted by the Research Ethics Committee of the University of Pretoria with protocol number 173/2022.

## Data Availability

The data that support the findings of this study are available on request from the corresponding author. The data are not publicly available due to privacy or ethical restrictions.
